# Coevolution of Sites under Immune Selection Shapes Epstein–Barr Virus Population Structure

**DOI:** 10.1093/molbev/msz152

**Published:** 2019-07-02

**Authors:** Fanny Wegner, Florent Lassalle, Daniel P Depledge, François Balloux, Judith Breuer

**Affiliations:** 1 Division of Infection & Immunity, University College London, London, United Kingdom; 2 Microbial Evolutionary Genomics, Institut Pasteur, Paris, France; 3 UCL Genetics Institute, University College London, London, United Kingdom; 4 MRC Centre for Outbreak Analysis and Modelling, Imperial College, London, United Kingdom; 5 Department of Microbiology, New York University School of Medicine, New York, USA

**Keywords:** Epstein–Barr virus, linkage disequilibrium, immune selection, virus-host coevolution

## Abstract

Epstein–Barr virus (EBV) is one of the most common viral infections in humans and persists within its host for life. EBV therefore represents an extremely successful virus that has evolved complex strategies to evade the host’s innate and adaptive immune response during both initial and persistent stages of infection. Here, we conducted a comparative genomics analysis on 223 whole genome sequences of worldwide EBV strains. We recover extensive genome-wide linkage disequilibrium (LD) despite pervasive genetic recombination. This pattern is explained by the global EBV population being subdivided into three main subpopulations, one primarily found in East Asia, one in Southeast Asia and Oceania, and the third including most of the other globally distributed genomes we analyzed. Additionally, sites in LD were overrepresented in immunogenic genes. Taken together, our results suggest that host immune selection and local adaptation to different human host populations has shaped the genome-wide patterns of genetic diversity in EBV.

## Introduction

Epstein–Barr virus (EBV) is a member of the gamma-herpesviruses family, present in most humans worldwide. Primary infection is either symptomless or causes infectious mononucleosis, and is followed by lifelong latent infection within the memory B cell pool. EBV has been associated with a variety of cancerous diseases of B cell origin, such as endemic Burkitt’s lymphoma (BL), Hodgkin’s lymphoma (HL), and posttransplant lymphoproliferative disorder (PTLD), cancers of epithelial origin including nasopharyngeal carcinoma (NPC) and gastric carcinoma and even in rare cases NK- and T-cell tumors (Young et al. 2016). Recent evidence also points toward an involvement in autoimmune diseases such as multiple sclerosis and systemic lupus erythematosus (Lossius et al. 2012; Pender and Burrows 2014). Development of disease is associated with various factors, such as immune status (e.g., PTLD, HIV-related lymphoma), coinfections (e.g., endemic BL and Malaria, HL and HIV) and geography with EBV-positive NPC particularly prevalent in adults from Southern China and Northern Africa, and endemic BL in children from equatorial Africa.

The double-stranded DNA genome of EBV has a length of around 172 kb and contains at least 94 annotated open reading frames (ORFs). It usually resides as a circular, double-stranded DNA molecule in the nucleus. Previous whole genome sequencing analyses have focused on geographically related strains and provided evidence for extensive recombination (Kwok et al. 2014; Palser et al. 2015). Palser et al. (2015) reported two cases of intertypic recombinants, but also presented evidence for multiple recombination events throughout the genome. This latter observation crucially impacts how EBV ancestry can be studied as recombination events are expected to affect tree topology and can render inference derived from phylogenetic approaches largely meaningless (Rieux and Balloux 2016).

Because pervasive recombination in EBV prevents us from inferring a single, genome-wide evolutionary history, we dissected the population structure of EBV genomes at the level of single nucleotides or genes. We adapted and applied a recently developed approach (Lassalle et al. 2016) based on genome-wide patterns of linkage disequilibrium (LD) to a data set comprising 223 EBV genomes collected from all around the world. Specifically, our analysis focuses on how sequence variation, recombination, and linkage have shaped the global population structure of EBV and may have influenced its evolution.

## Results

### EBV Genome Sequences Are Highly Recombinant

We utilized a data set comprising 223 type 1 EBV whole genome sequences that have previously been published (de Jesus 2003; Zeng et al. 2005; Liu et al. 2011; Kwok et al. 2012; Lin et al. 2012; Lei et al. 2013; Tsai et al. 2013; Kwok et al. 2014; Palser et al. 2015; Chen et al. 2018; Correia et al. 2018; Bridges et al. 2019; Hui et al. 2019). These samples are representative of diverse geographical regions, body compartments, and malignancies (supplementary table 1, Supplementary Material online).

We first examined our data set for evidence of recombination. A PHI-test (Bruen et al. 2006) found global evidence for recombination (*P* < 0.05) and a genome-wide PHI-profile scan revealed areas of significant recombination throughout the genome (supplementary fig. 1, Supplementary Material online). The presence of numerous reticulations in a recombination network confirms this (supplementary fig. 2, Supplementary Material online). Consequently, genetic recombination cannot be ignored, thus precluding the use of phylogenetic inference from whole genome sequences.

### Evidence of Genome-Wide LD despite Widespread Recombination

A useful way to assess recombination on a larger scale is to consider LD, that is, the correlation between the occurrence of polymorphisms at different loci in the genome (Haydon et al. 2004). Two loci are considered to be in LD when they occur together more often than would be expected by chance under a uniform distribution of allele combinations given their respective frequencies. There are several factors influencing LD, including physical proximity, the rate of recombination, natural selection, and population structure. For a given recombination rate, the likelihood of a recombination event is inversely proportional to the physical distance between a pair of loci, a negative relationship is expected between the LD between two biallelic sites and the physical distance separating them.

**Fig. 1.**
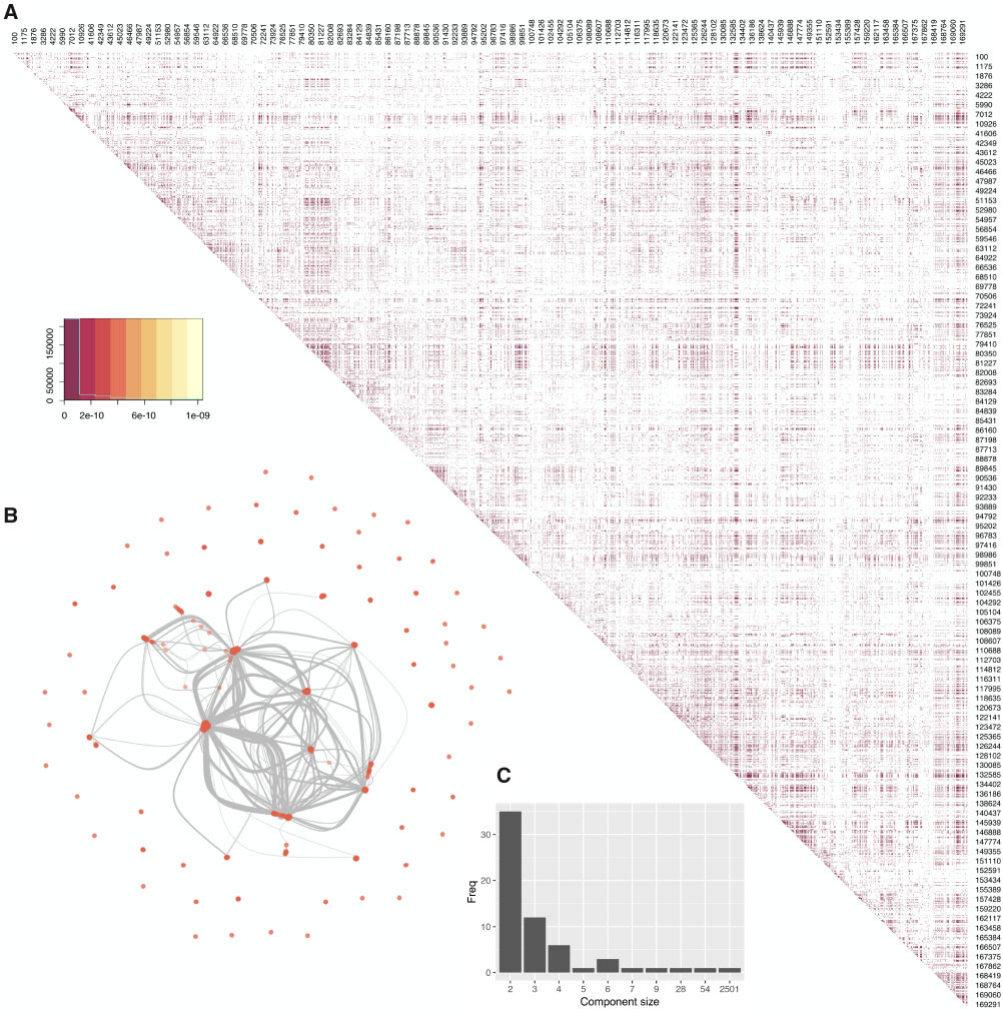
(*A*) Heatmap of SNPs which are in significant LD. Darker colors indicate lower *P* values, with insignificant pairs being colors white. Numbers denote the genome positions (sampled uniformly), with rows and columns that did not contain any significant pair of SNPs in LD removed. (*B*) Association network of all sites in LD with at least one other site. Each node represents a biallelic site, each link between two nodes signifies they are in LD with each other. (*C*) Frequency plot of all connected components in the association network.

Genome-wide LD was assessed for all combinations of biallelic sites using Fisher’s Exact test (significant if *P* < 0.05 after Bonferroni correction). Figure 1*A* shows a map of LD between biallelic sites. In total, 253,935 pairs of sites were found to be in LD, which represents 2,752 individual sites out of the 9,822 biallelic sites analyzed. There are 242,372 pairs with at least one site being located in an ORF and 165,407 pairs where both sites fall within ORFs (2,229 unique sites). Of these, there are 41,587 pairs where allelic variation at both sites is synonymous, 81,582 pairs with at least one nonsynonymous site and 42,238 pairs where allelic variation at both sites correspond to nonsynonymous changes.

LD was essentially independent of physical distance in the genome between linked sites (supplementary fig. 3, Supplementary Material online), that is, with similar distributions of *P* values over many distance classes, although proximal sites showed a lower distribution of *P* values. The detection of LD throughout the genome even when sites are distal (fig. 1*A*) is somewhat counterintuitive given the evidence of pervasive recombination. Focusing on subsets of sites that are in LD with at least one other site (all sites in LD, nonsynonymous sites in LD, and synonymous sites in LD), recombination networks (supplementary fig. 4, Supplementary Material online), and PHI-test still gave evidence for recombination occurring within all subsets (*P* < 0.05). Figure 1*B* shows an association network of sites linked with each other. Each site is represented by a circle, while connections are drawn between them if they are in LD. We recovered 62 components, that is, subnetworks in which every pair of sites (nodes) is connected by a path. The components consisted of a large network comprising the majority of sites (2,501 out of 2,752, fig. 1*C*), and smaller sets and pairs of independently linked single nucleotide polymorphisms (SNPs) (fig. 1*B* and *C*). However, even the largest component displays evidence for recombination (supplementary fig. 5, Supplementary Material online). For the subsequent analyses we focused on the largest component of the network, referred to as the major component, to provide a majority, nonchimaeric representation of the genome’s linkage structure.

### Genome-Wide LD Can Be Explained by Population Structure

One possible explanation for the pattern of linkage is the influence of population structure whereby apparent coinheritance of biallelic sites simply reflects the independent segregation of different alleles in isolated populations. We used the program Admixture (Alexander et al. 2009) to cluster individuals into populations. We found no evidence for genetic subdivision by body compartment and malignancy (results not shown).

**Fig. 2.**
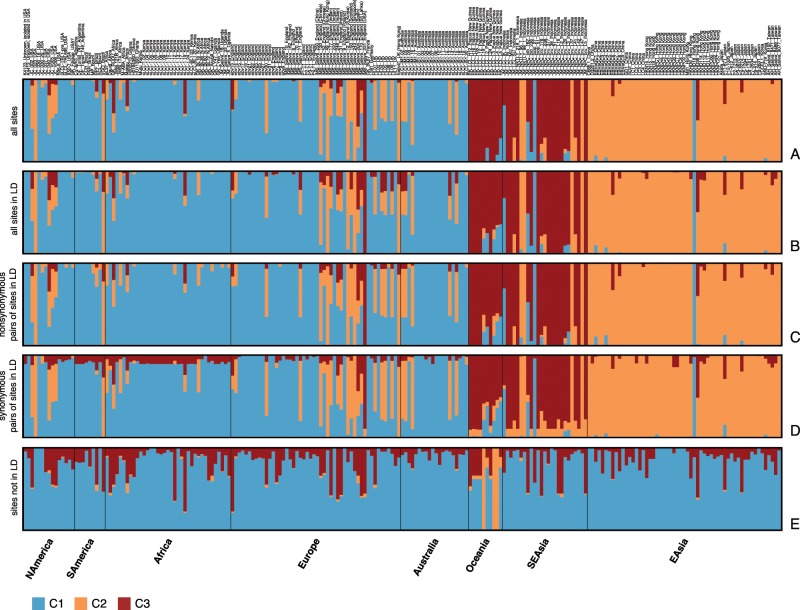
Population assignment for all genome sequences assuming a population number of *k *=* *3 for different subsets of sites. Every bar represents a strain that has been preassigned to either “Africa,” “Asia” or “Western” (comprised of American, European, and Australian isolates). The coloring of the bars represents the proportion of the input sites that have been assigned to a certain population. (*A*) all biallelic sites; (*B*) all sites in LD in the largest component; (*C*) nonsynonymous pairs of sites in LD; (*D*) synonymous pairs of sites in LD; (*E*) sites not in LD. (*B*–*D*) refer to the subset of sites in LD that are in the largest component in the association network (fig. 1*B*).

Using all biallelic sites (fig. 2*A*), a very striking top-level structure could be uncovered when assuming three subpopulations. Almost all sequences originating from Asia and Oceania were assigned to two clusters (C2 and C3, dark red and orange), whereas the majority of African, European, North and South American as well as Australian isolates belong to a third cluster (C1, blue), suggesting the existence of two separate virus populations in East Asia and in the Pacific, as well as a third separate population of viruses spread throughout the rest of the world (as represented by the data set). A number of sequences could not be unambiguously assigned to a single cluster and are likely of admixed ancestry.

Restricting the data set to all biallelic sites in LD (major component) (fig. 2*B*) did not change the proportional assignment of isolates to populations. The same remains true when the analysis was restricted to the subset of pairs on nonsynonymous and synonymous sites in LD (fig. 2*C* and *D*). By contrast, biallelic sites not in LD with any other site do not show evidence for a clear population structure corresponding to any geographic pattern, as the assignment to C3 and C1 is noisy across genomes (fig. 2*E*), with the exception of three samples from Oceania (all from Papua New Guinea). These three sequences are on very long branches in the recombination network (supplementary fig. 2, Supplementary Material online) indicating they are very distant from the rest of the data set. The sites responsible for these long branches were probably not detected as being in LD due to the low number of sequences in which they occur.

This top-level view does not capture the full complexity of the EBV population structure. We inferred that clustering the isolates into 20 subpopulations best describes the data (supplementary fig. 6, Supplementary Material online). This leads to an increasingly finer structure in each of the geographical groups. There is a large overlap in assignment between sequences from Europe and Australia as well as some from North America. By contrast, there are a few clusters formed only by African sequences, highlighting the subtler differences between genomes from Africa and Europe/Australia. Similarly, a finer structure within the Southeast Asian, East Asian, and Oceanian sequences emerges. For example, there are distinct subpopulations within China and Hong Kong, and one subpopulation frequently associated with Japan. Although several of these additional subpopulations are comprised of unadmixed individuals, a large number of sequences are strongly admixed, confirming the persistent signal of recombination even within the subset of sites in LD (supplementary figs. 4–5, Supplementary Material online).

### The Population Structure of EBV Is Linked to Immune Genes

**Fig. 3.**
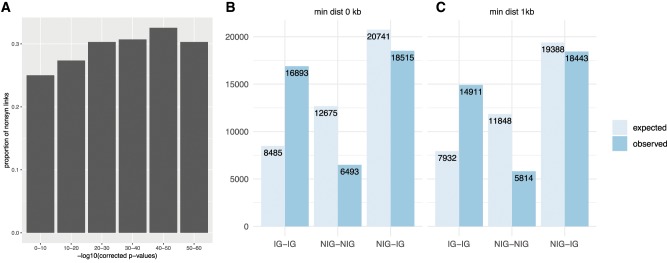
(*A*) Proportion of pairs of nonsynonymous sites in LD with each other over LD strength. (*B*–*C*) Number of links between nonsynonymous sites between different categories of genes. (*B*) All sites (chi-square test, *P* < 2.2e-16). (*C*) Sites with a minimal distance of 1 kb (chi-square test, *P* < 2.2e-16).

Selection acts primarily on polymorphisms resulting in amino acid changes, and selection for cofunctional substitutions in proteins could explain the observed LD pattern. In this context, it is interesting to note that the proportion of nonsynonymous sites in linkage increases with the strength of LD (fig. 3*A*). To determine which genes are preferentially linked to each other, the data were restricted to nonsynonymous sites. When examining which genes contained sites that are most often found in LD (number of SNPs in LD), the genes in the top 1% of gene pairs were *BPLF1*, *BOLF1*, *BLLF1*, *EBNA3A-C*, *EBNA1*, *BcRF1*, and *LMP1* (supplementary fig. 7, Supplementary Material online). Interestingly, seven of these nine ORFs are known to encode antigens. The other two genes (*BcRF1* and *BPLF1*) do not fulfill our conservative criterion of carrying at least two independent records of experimentally confirmed epitopes.

This led us to hypothesize that adaptation to the host immune system and maintenance of a variation at a specific subset of sites might have played a role in shaping the global population structure of EBV. To test for this, the data set of genes was divided in two: genes that are known to code for immunogenic (IG) proteins and nonimmunogenic (NIG), for which there is no current record of experimentally confirmed epitopes (table 1). Nonsynonymous sites within these ORFs belonging to IG are more often in LD with each other than would be expected if a uniform distribution of links across all genes is assumed (*P* < 2.2e-16, chi-square test), even when excluding links between proximal SNPs (fig. 3*B* and *C*). Conversely, genes belonging to NIG are less often linked with each other than expected by chance.

**Table 1.** List of 33 Genes Present in the Gene Network Considered to Code for Immunogenic Proteins.

Protein	ORF	Number of Epitopes
Major DNA-binding protein	*BALF2*	2
Tripartite terminase subunit UL28 homolog	*BALF3*	1
Envelope glycoprotein B	*BALF4*	16
DNA polymerase catalytic subunit	*BALF5*	1
Ribonucleoside-diphosphate reductase small chain	*BaRF1*	1
Portal protein UL6 homolog	*BBRF1*	1
Major capsid protein	*BcLF1*	3
Triplex capsid protein VP23 homolog	*BDLF1*	1
Capsid protein VP26	*BFRF3*	13
Protein BGLF3	*BGLF3*	1
Apoptosis regulator BHRF1	*BHRF1*	3
Envelope glycoprotein GP350	*BLLF1*	2
Deoxyuridine 5′-triphosphate nucleotidohydrolase	*BLLF3*	3
DNA polymerase processivity factor BMRF1	*BMRF1*	8
Protein BMRF2	*BMRF2*	1
Major tegument protein	*BNRF1*	4
Protein BOLF1	*BOLF1*	2
Ribonucleoside-diphosphate reductase large subunit	*BORF2*	1
Replication and transcription activator	*BRLF1*	8
Tegument protein BRRF2	*BRRF2*	2
DNA primase	*BSLF1*	1
Envelope glycoprotein H	*BXLF2*	11
Transactivator protein BZLF1	*BZLF1*	24
Epstein–Barr nuclear antigen 1	*EBNA1*	82
Epstein–Barr nuclear antigen 2	*EBNA2*	12
Epstein–Barr nuclear antigen 3	*EBNA3A*	30
Epstein–Barr nuclear antigen 4	*EBNA3B*	23
Epstein–Barr nuclear antigen 6	*EBNA3C*	33
Protein LF2	*LF2*	1
Uncharacterized protein LF3	*LF3*	1
Latent membrane protein 1	*LMP1*	17
Latent membrane protein 2	*LMP2*	32

Note.—Each epitope must have at least two references listed in IEDB.

As sites are linked with each other across the whole genome, that is, SNPs (and ORFs) are not only linked to one but to several other SNPs (and ORFs), we sought to study this interconnectedness with a graph theoretical approach at the level of individual genes. The resulting gene network consisted of 73 genes, 32 of them belonging to IG and 41 belonging to NIG, respectively. Edges were weighted based on a linkage score. This linkage score was significantly higher for edges between genes both belonging to IG (Mann–Whitney *U* test, *P* = 0.005 for IG–IG vs. NIG–NIG, and *P* = 3.6e-5 for IG–IG vs. NIG–IG, respectively; supplementary fig. 8, Supplementary Material online). Structurally, this network is connected, that is, any node can be reached by any other node through one or more edges (there are no disconnected components). However, structure within the network in terms of clustering seems to be low.

**Fig. 4.**
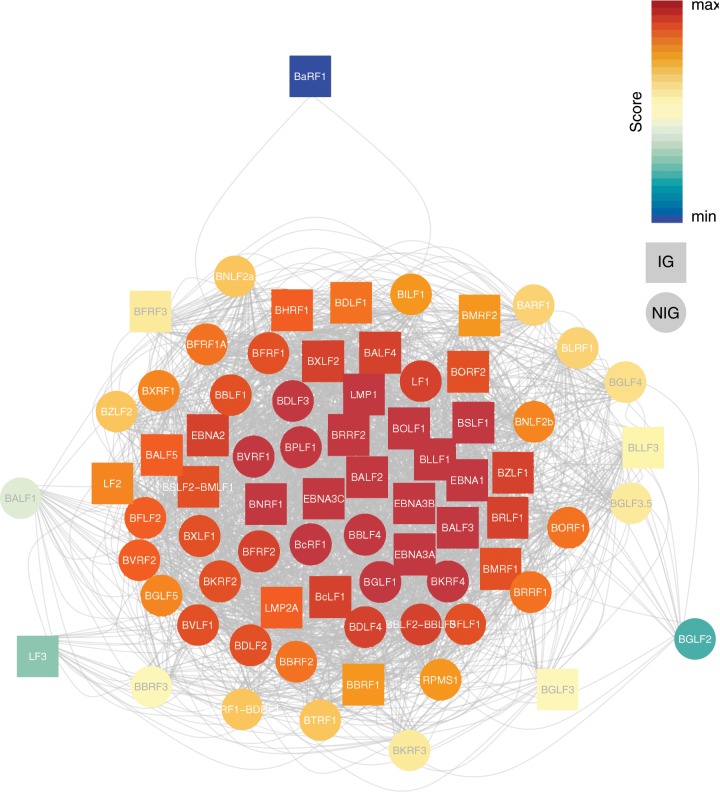
Whole gene network, colored based on Eigenvector centrality, with warm colors indicating higher and cooler colors lower scores, respectively. Square node symbols denote genes belonging to IG, circular nodes denote genes belonging to NIG.

Identifying the most important ORFs in a network can be done by ranking nodes based on their properties. Eigenvector centrality does this by measuring the influence of a node, that is, a node’s score is higher if it is connected to other high-scoring nodes (fig. 4). Of the top 25 highest ranked genes, 11 belong to the IG group (table 2). An additional five genes within the top 25 also appear in the IEDB database as antigens, but did not meet our conservative criterion of a minimum of two independent records as experimentally confirmed antigens. In total, 36 of the 94 annotated ORFs in the EBV genome contain experimentally confirmed epitopes that fulfill this criterion. Of those, 32 are represented in the linked gene network (table 1), and 11/32 IG nodes are within the top 25 highest ranked genes.

**Table 2.** Most Influential Nodes in the Network.

Eigenvector Rank	ORF	Protein	IG
1	*BPLF1*	Large tegument protein deneddylase	○
2	*BcRF1*	TBP-like protein	
3	*BBLF4*	DNA replication helicase	
4	*EBNA3B*	Epstein–Barr nuclear antigen 4	●
5	*BLLF1*	Envelope glycoprotein GP350	●
6	*BNRF1*	Major tegument protein	●
7	*EBNA1*	Epstein–Barr nuclear antigen 1	●
8	*BALF3*	Tripartite terminase subunit 1	
9	*BGLF1*	Capsid vertex component 1	○
10	*BKRF4*	Tegument protein	
11	*EBNA3A*	Epstein–Barr nuclear antigen 3	●
12	*LMP1*	Latent membrane protein 1	●
13	*EBNA3C*	Epstein–Barr nuclear antigen 6	●
14	*BOLF1*	Protein BOLF1	●
15	*BRRF2*	Tegument protein	○
16	*BALF2*	Major DNA-binding protein	
17	*BDLF3*	BDLF3 (Glycoprotein)	
18	*BSLF1*	DNA primase	
19	*BVRF1*	Capsid vertex component 2	○
20	*BRLF1*	Replication and transcription activator	●
21	*BXLF2*	Envelope glycoprotein H	●
22	*BDLF4*	Uncharacterized protein	
23	*LF1*	Uncharacterized protein	
24	*BBLF2–BBLF3*	DNA helicase/primase complex-associated protein	○
25	*BALF4*	Envelope glycoprotein B	●

Note.—Circles in the column labeled IG mark proteins for which an immune response has been reported, with filled circles fulfilling the criterium of having at least two references and empty circles having fewer than two.

## Discussion

Recombination plays an important role in viral evolution as a source of genetic diversity. By combining mutations that previously appeared separately in different genomes, recombination allows the creation of new haplotypes. Genome-wide recombination has been described in detail for all well-studied herpesviruses (Norberg et al. 2007; Smith et al. 2013; Norberg et al. 2015; Lassalle et al. 2016; Koelle et al. 2017). It has been best studied at the molecular level in herpes simplex virus 1 (HSV-1) where it is part of the replication process (Wilkinson and Weller 2003). However, recombination has also been proposed as a mechanism for herpesviruses to maintain the integrity of viral genomes during latency (Wilkinson and Weller 2004; Brown 2014).

Here we show that despite extensive recombination, EBV retains considerable population structure with evidence for extensive genome-wide LD. These findings differ from those observed for human cytomegalovirus (HCMV) (Lassalle et al. 2016) and HSV-1 (Lassalle et al. unpublished data) both of which appear to be essentially freely recombining with only localized areas of LD. The extent to which free recombination can occur in different herpesviruses might be due to their employment of the host’s homologous recombination (HR) systems (Brown 2014).

In alpha-herpesviruses such as HSV-1 and Varicella zoster virus, inverted and tandem repeats are the most prevalent HR initiating sequences and these are enriched in the unique short (U_S_ or S) segment of the genome. By contrast, HR initiating sequences in gamma-herpesviruses like EBV have been shown to be specific short GC-rich sequences that are evenly distributed across the genome (Brown 2014). Additional studies are required to better understand these apparent differences in LD and recombination across different herpesvirus families. Other potential causes for disparities in the inferred recombination intensity between these viral species may be driven by their opportunity to recombine and the distribution of the genetic divergence of recombining viral strains.

In the presence of extensive genetic recombination, LD is generally driven by an underlying population structure, which in turn might reflect biological or environmental constraints (McVean et al. 2002), or genetic drift within geographically segregated populations. In the case of EBV, population structure analyses identified a complex structure which largely corresponds on the top-level to three geographic groups: East Asia, Oceania (represented by Papua New Guinea) and Southeast Asia (represented by Indonesia), and the rest of the world. This structure is primarily supported by the sites in LD, with the exception of three very divergent sequences from Oceania (supplementary fig. 2, Supplementary Material online) for which there is a signal of structure even in the set of SNPs not in LD. On a finer scale, EBV population structure is naturally more complex. In our analysis, we found 20 subpopulations to best describe our data. Similarly, a previous study with a smaller, partly different data set (including Mediterranean and type 2 sequences) described ten subpopulations (Chiara et al. 2016). Both works show a correlation between geography and subpopulation, in particular with distinct subpopulations present in Africa, as well as overlapping subpopulations across the world, for example, Europe/Australia and America/Africa. However, our study also highlights the presence of distinct subpopulations within Asia (and Oceania), which was previously not described.

Our data confirm previous findings where Asian and Indonesian sequences cluster quite distinctly from other genomes in a PCA (Palser et al. 2015; Correia et al. 2018). A few samples have been assigned to different clusters than the majority of genomes from the same region. For example, one genome from Hong Kong and one genome from Indonesia have been completely assigned to C1 (associated with non-Asian and non-Oceanian sequences). Similarly, four sequences from Indonesia, one sequence each from the United States and from Brazil, as well as five United Kingdom sequences seem to belong to C2 (associated with East Asia). However, the geographic label of the sequences is based on where they have been isolated and does not necessarily reflect the actual evolutionary origin of the virus genotype. There are only a handful of sequences isolated in the United Kingdom, for which the geographic origin of the donor is given (mentioned in brackets in fig. 2 and supplementary table 1, Supplementary Material online) and where the assignment to different clusters than C1 is traceable. It is easily imaginable that a sequence isolated in the United Kingdom or the United States, countries with mixed ethnic populations, could originally be an Asian strain, as primary infection often occurs through close family members in early age (Hjalgrim et al. 2007).

Recombination is also occurring within these subpopulations, with evidence for recombination within the subset of sites in LD (PHI-test, *P* < 0.05). Within-population recombination is also supported by the presence of admixed individuals in the population structure assignment, both for the top-level structure as well as the fine-resolution population structure analysis into 20 subpopulations (supplementary fig. 7, Supplementary Material online). HKNPC2, for example, has been described as recombinant of HKNPC7 and -9 (Kwok et al. 2014), all isolates from Hong Kong that have been clearly assigned to the East Asian population (Palser et al. 2015).

In contrast to other herpesviruses, and despite this ever-present signal of recombination between and within subpopulations, EBV largely maintains its population structure. Interestingly, the proportion of nonsynonymous sites increases with the strength of LD (fig. 3*A*). This finding suggests that the pattern of genetic linkage is driven by natural selection on encoded proteins, notably preserving combination of residues determining how proteins functionally interact with each other. This hypothesis is compatible with the correlation of higher strength of LD with the increase in fraction of nonsynonymous biallelic sites and could indicate that synonymous sites are less likely to be constrained to coevolve; instead, the existence of stronger LD within the 2 kb range (supplementary fig. 3, Supplementary Material online) suggests that synonymous sites may be hitch-hiking with physically linked sites, that is, only contingently segregating with closely located nonsynonymous sites under selection.

The association of linked polymorphism distribution with geography or ethnicity might reflect different biological constraints in each viral subpopulation. We thus investigated the possibility that important protein–protein interactions (PPI) could be determining EBV population structure. By representing the genes with the strongest LD in a linked gene network (fig. 4), we were able to identify the 25 most important nodes via an Eigenvector centrality-based ranking and compare them with data on PPIs previously described for EBV (Calderwood et al. 2007; Fossum et al. 2009) (supplementary fig. 9, Supplementary Material online). Although a few recovered interactions relate to known interactions (e.g., interaction between tegument and envelope proteins BPLF1, BALF4, and BOLF1), no straightforward hypothesis of biological cause can be proposed for others, due to their primary expression occurring in different stages of the life cycle as well as the current evidence for their localization within the cell or virion, for example between the proteins encoded by BDLF3 (a glycoprotein expressed late in lytic cycle) and EBNA3A (which is located in the nucleus and expressed during latency). Nevertheless, PPI might explain some of the sites in LD observed in NIG genes.

Since PPI data based on yeast two hybrid screen are known to have a high false positive rate (Deane et al. 2002), we looked for other explanations for gene associations and considered a possible role of variation in host genetic makeup. Host genetics has been previously suggested to shape pathogen population structure, for example in HIV (Kløverpris et al. 2016), *Mycobacterium tuberculosis* (Gagneux et al. 2006; Gagneux 2012) or *Helicobacter pylori* (Thorell et al. 2017). However, it is challenging to disentangle the effect of the demography of the pathogen and its host(s) from local adaption of a pathogen to its different host populations and their wider environment. It is probably fair to state that so far there is no case where host genetics could be uncontroversially identified as the driver for the apportionment of genetic diversity in a pathogen. Moreover, none of the previous putative cases of within-species host adaptations invoked selective pressures at such a large number of variants spread throughout the genome of a pathogen. Our results on EBV are also to the best of our knowledge the first case of such widespread gene-by-gene epistasis.

In EBV, the number of LD links between genes encoding proteins that are targets of adaptive immunity was significantly higher than between proteins with no adaptive immune function (fig. 3*B* and *C*). Moreover, 11 out of the 25 most important linked genes code for protein sequences that are the target of adaptive immunity. However, some of the genes we classify as NIG might in fact also contain epitopes that have not been described so far. The results raise the possibility that the EBV population structure may to a large extent have been shaped by host immunity, perhaps because the virus has adapted to HLA alleles common in the subpopulation in which it is circulating. The data are in concordance with a model of nonoverlapping combinations of epitope regions, that are being held in LD despite genetic exchange via recombination between pathogens in other parts of the genome (Gupta et al. 1996).

In summary, we find that EBV retains a strong population structure in the face of considerable recombination and that this population structure is geographically stratified. The maintenance of the viral population structure may be partly driven by intrinsic viral coadaptation of genes. Though, the evidence that genes in strongest LD are enriched in IG genes suggests that adaptive immune selection, likely HLA mediated, has played a significant role in the maintenance of epistasis within this population. This raises the intriguing possibility that host genetic factors of the human populations in which the virus subpopulations have been circulating have been shaping the global population structure of EBV through local adaptation to its local human host populations. Irrespective of the underlying evolutionary forces, our findings starkly distinguish EBV from other human herpesviruses such as HCMV, which has been shown to be essentially freely recombining (Lassalle et al. 2016).

## Materials and Methods

### Data Set

The data set consisted of 223 type 1 EBV whole genome sequences available from GenBank (supplementary table 1, Supplementary Material online), comprising samples from various geographical regions and malignancies. The selection of sequences is explained in the Supplementary Material online.

### Sequence Analysis

Multiple sequence alignments were obtained using mafft v7.407 (Katoh and Standley 2013) and manually corrected to reduce unnecessary gaps generated around short tandem repeat sequences. SNPs were called based on differences to the reference genome NC_007605 (B95-8) using the R packages adegenet (Jombart 2008) and ape (Paradis et al. 2004).

### Recombination and LD

A test for the presence of recombination (PHI-test) was performed on the whole genome alignment excluding gapped sites with PhiPack under default parameters (Bruen et al. 2006). Additionally, a profile of PHI-test *P* values was computed along the genomes with sliding window of 1,000 bp and a step size of 25 bp. Split networks were generated using Splitstree4 (Huson and Bryant 2006).

For the genome-wide analysis of linkage, the whole genome alignment of type 1 sequences was restricted to its 9,822 biallelic sites where maximally five sequence were missing. Linkage between all possible combinations of biallelic SNPs was tested with Fisher’s Exact test, with a pair of SNPs being significantly associated if *P* < 0.05 after Bonferroni correction. Based on all linked sites, an association network was constructed with igraph (Csárdi and Nepusz 2006).

Split networks were generated from subsamples of aligned sites: all SNPs, all linked SNPs (sites in LD with at least one other site), all linked sites of the biggest component of the association network, all nonsynonymous linked SNPs, and all synonymous linked SNPs.

### Population Structure

The population structure was analyzed with Admixture 1.3.0 (Alexander et al. 2009). It was run ten times for every *k* ranging from 1 to 40 with the cross-validation option to infer the number of clusters *k* that best fits the data. Replicate runs were further processed using CLUMPAK (Kopelman et al. 2015) and results for the major modes were visualized with Distruct (Rosenberg 2004).

### Gene Linkage Network

A network was constructed and analyzed with the R package igraph (Csárdi and Nepusz 2006), where each node represents an ORF. An edge between two nodes was drawn if there was at least one pair of nonsynonymous SNPs in LD between them. The edges were weighted by a linkage score based on the number of linked nonsynonymous SNPs between two ORFs, normalized by the sum of ORF lengths (expressed as a fraction of the genome length).

The nodes of the network were divided into two sets of nodes, (1) those ORFs known to encode antigens (IG) and (2) those that do not (NIG). This classification was based on the experimentally confirmed antigens found in the IEDB database (http://www.iedb.org, April 2019) (Vita et al. 2018), with restriction to those antigens whose epitopes had been confirmed by at least two studies.

## Supplementary Material

Supplementary data are available at *Molecular Biology and Evolution* online.

## Supplementary Material

msz152_Supplementary_Data
