# Supplementary material for: Coevolution of Sites under Immune Selection Shapes Epstein–Barr Virus Population Structure
Source: Mol Biol Evol. 2019 Jul 2;36(11):2512–21. doi: 10.1093/molbev/msz152 (PMC6805225; doi:10.1093/molbev/msz152)
Supplement: msz152_Supplementary_Data [file msz152_supplementary_data.zip › supplementary_material.pdf]

Supplemental figures

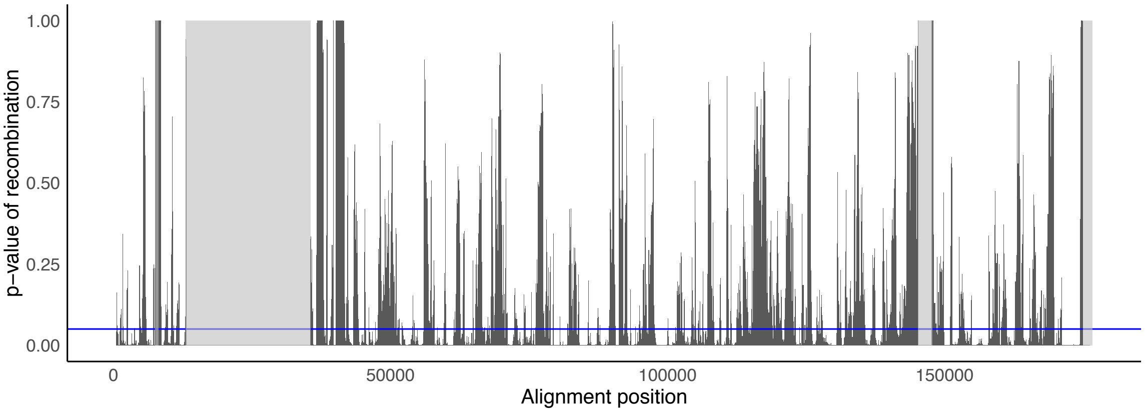

Suppl. Figure 1: Profile plot of p-values of the PHI-test across the genome. The blue line marks the significance threshold of alpha = 0.05. Repeat regions are marked in grey.

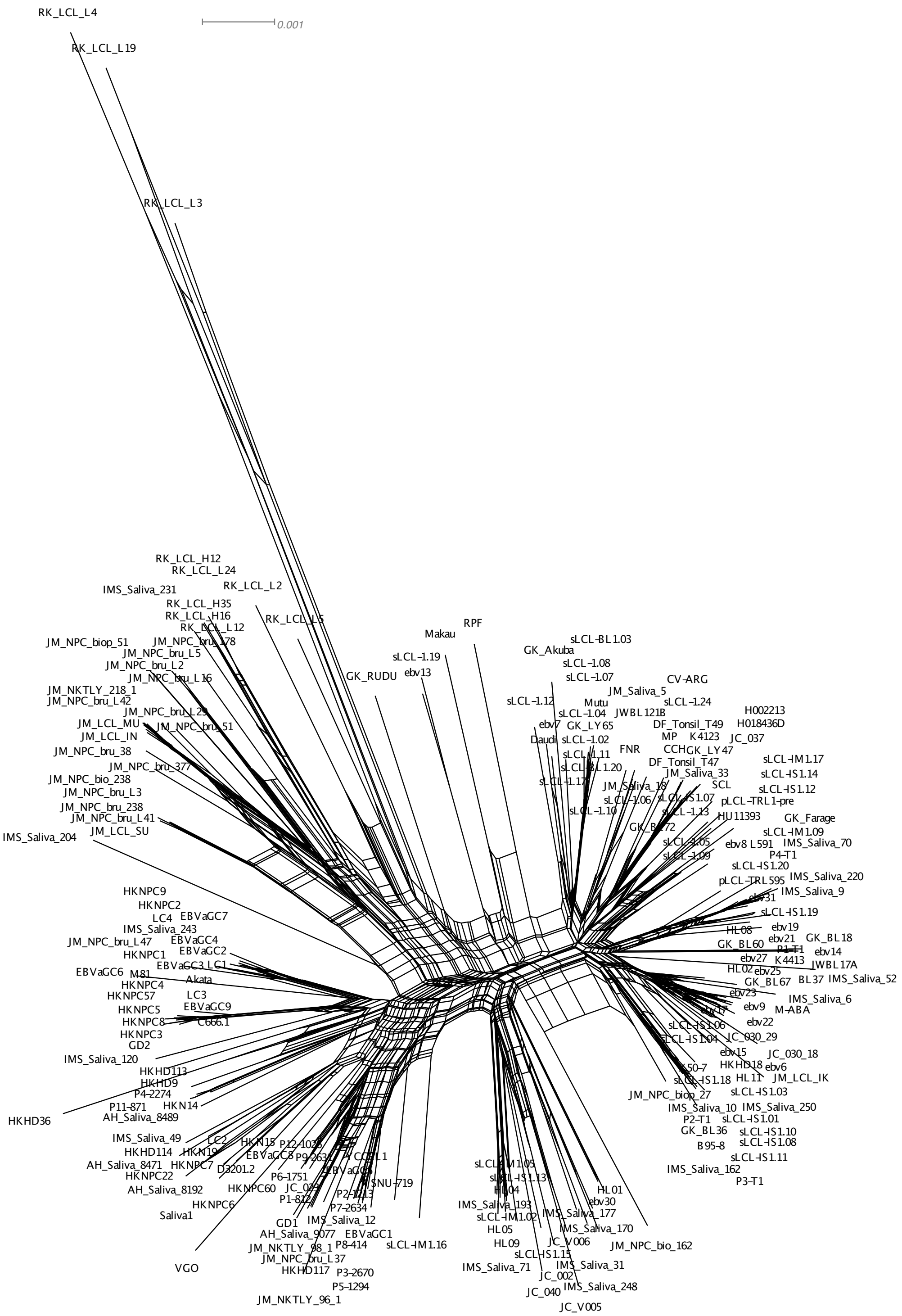

Suppl. Figure 2: Recombination network of the whole genome alignment of type 1 sequences.

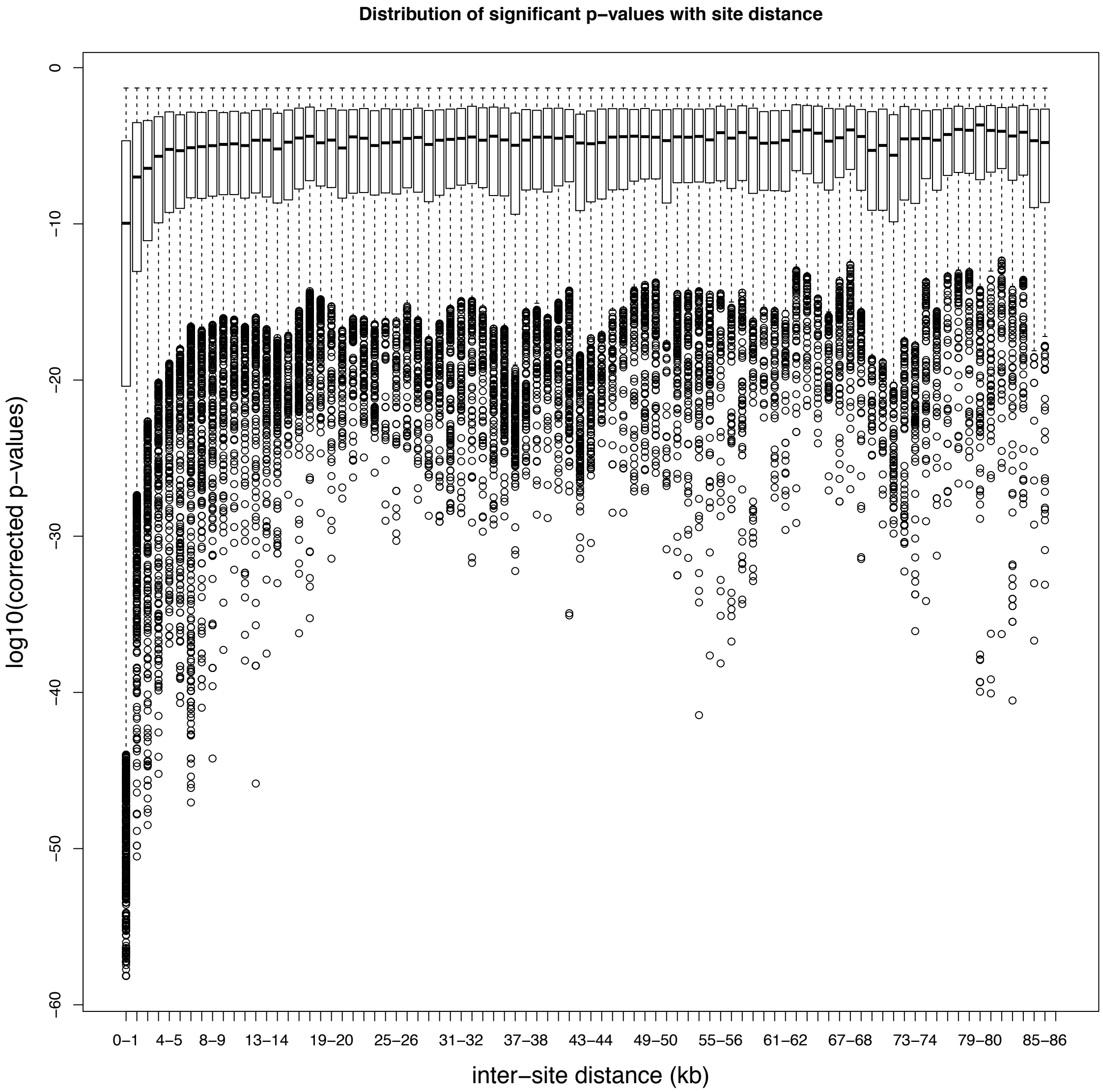

Suppl. Figure 3: Distribution of p-values of Fisher’s Exact test for all pairs in LD over site pair distance. The distance between two sites in LD have been binned into distance classes of 1kb size.

Suppl. Figure 4: Recombination networks of

A) all sites in LD,

B) all nonsynonymous sites in LD,

C) all synonymous sites in LD.

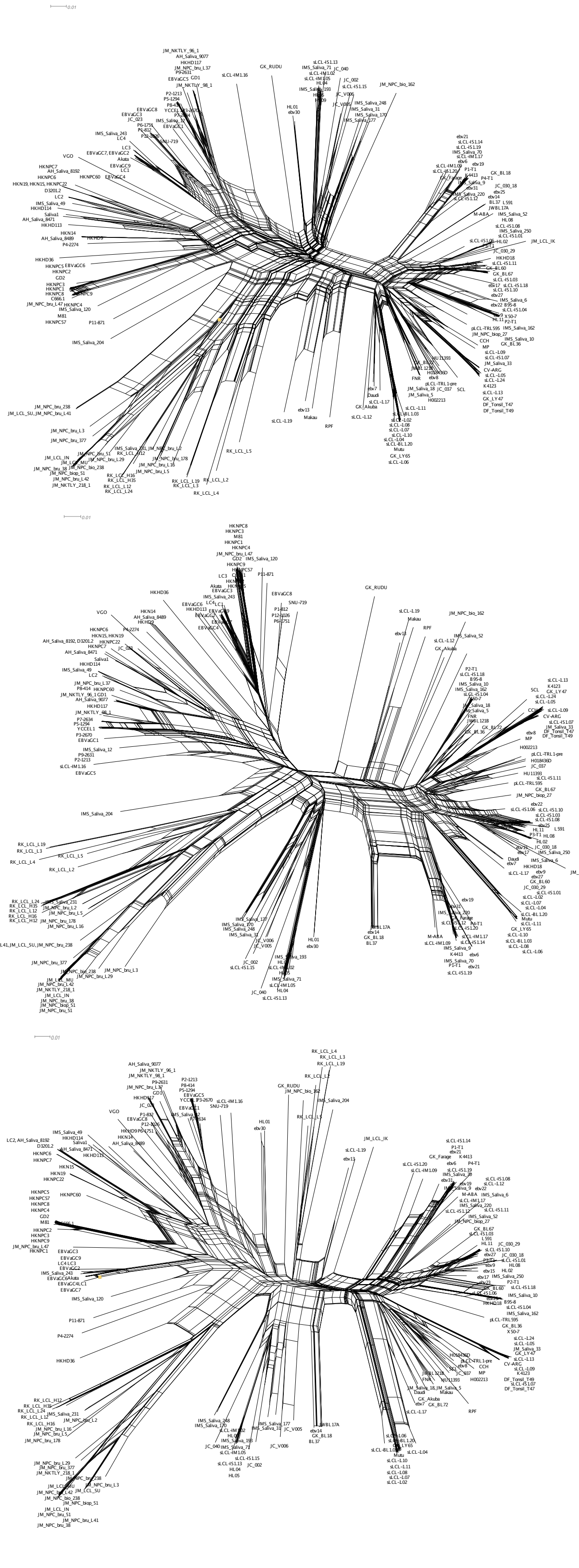

A

B

C

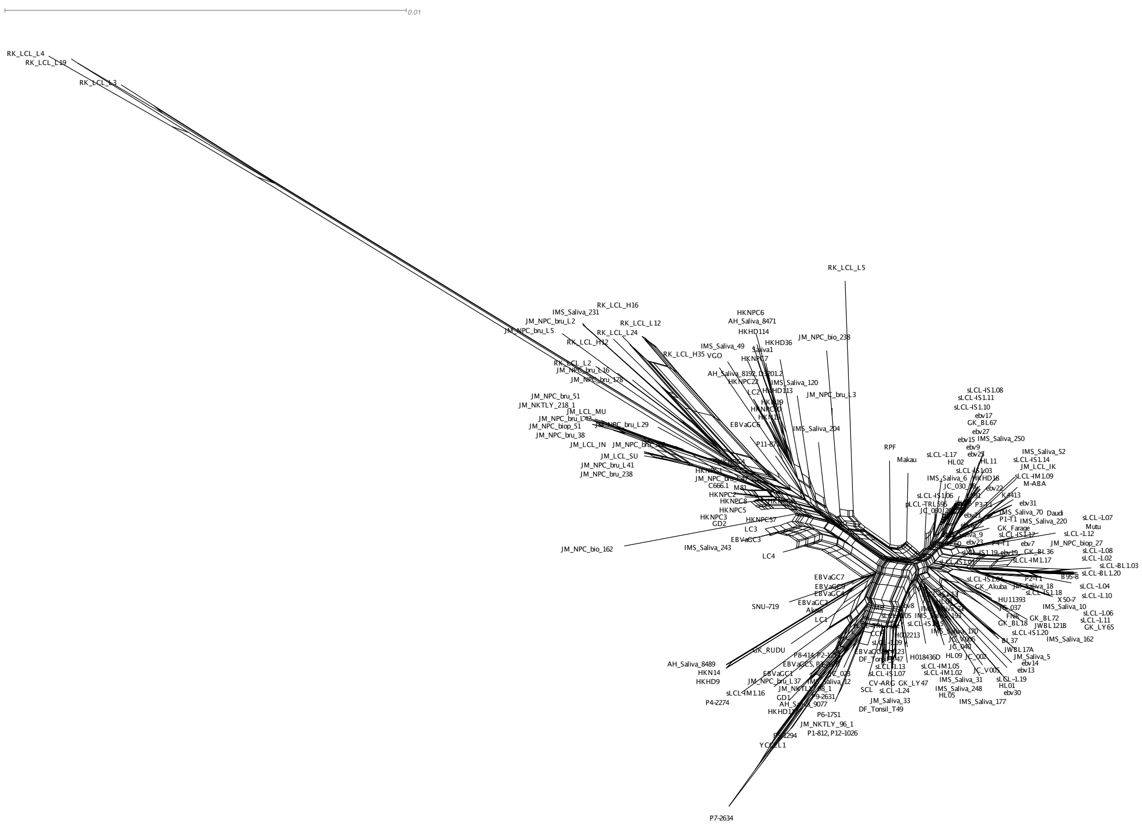

Suppl. Figure 5: Recombination network of the largest component in the association network of sites in LD (figure 1B).

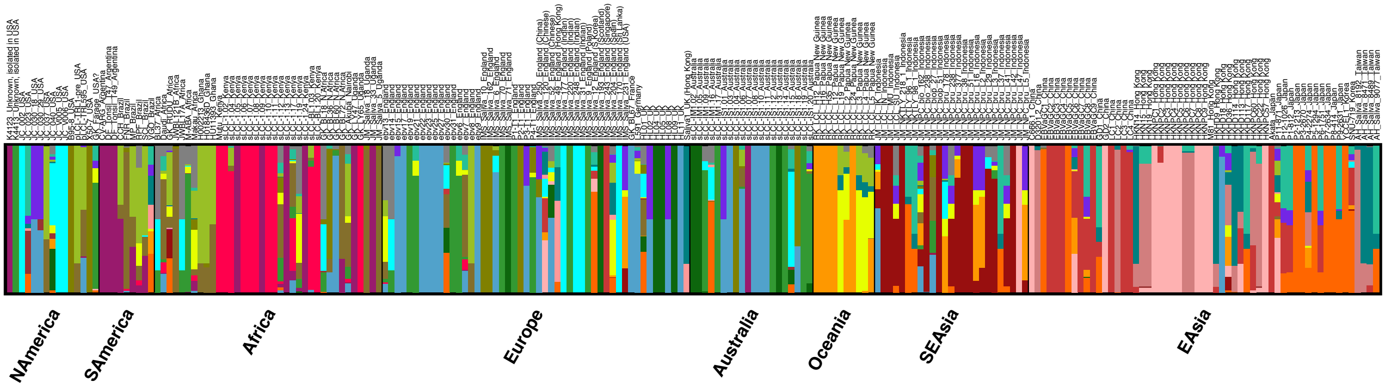

Suppl. Figure 6: Population assignment for all sites in LD assuming a population number of *k*=20.

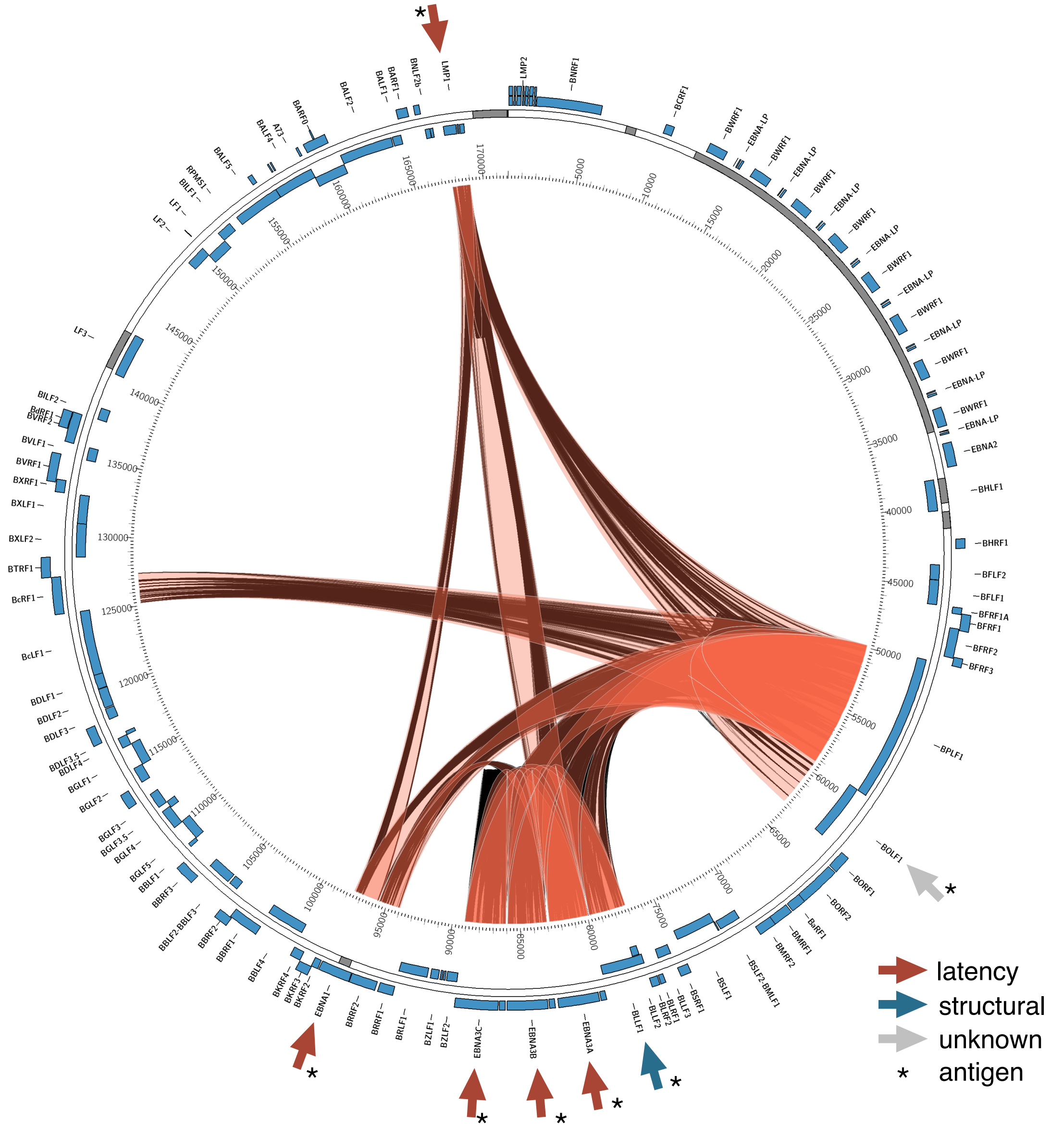

Suppl. Figure 7: Genome map depicting the EBV ORFs in blue on the outside and the repeat regions in grey (excluded from analysis). The connection across the genome mark the top 1% of ORFs that are most often linked via nonsynonymous sites. The red ribbon outlines the ORF, while the individual black lines mark the specific pairs of sites in LD.

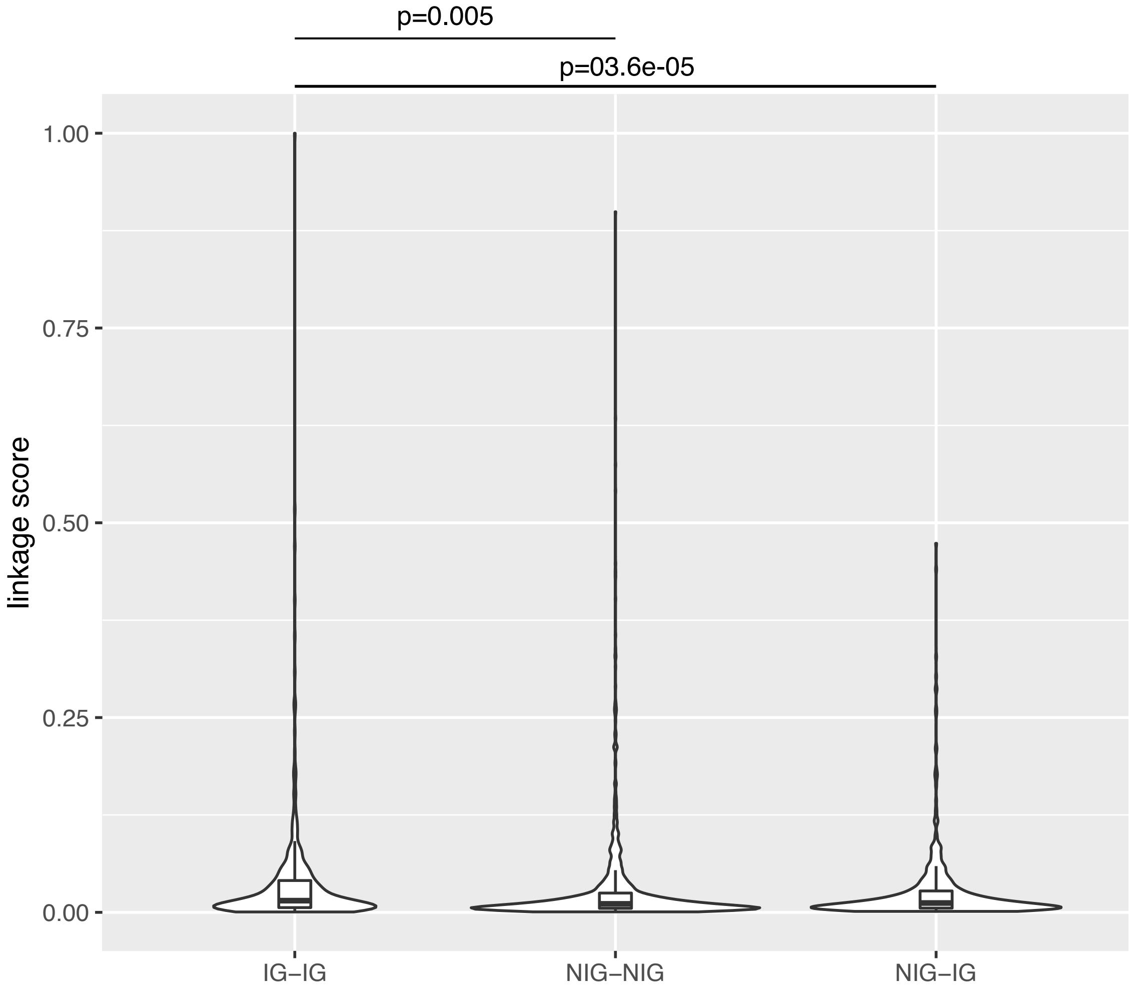

Suppl. Figure 8: Boxplot of linkage score (edge weight) between nodes belonging to different gene categories.

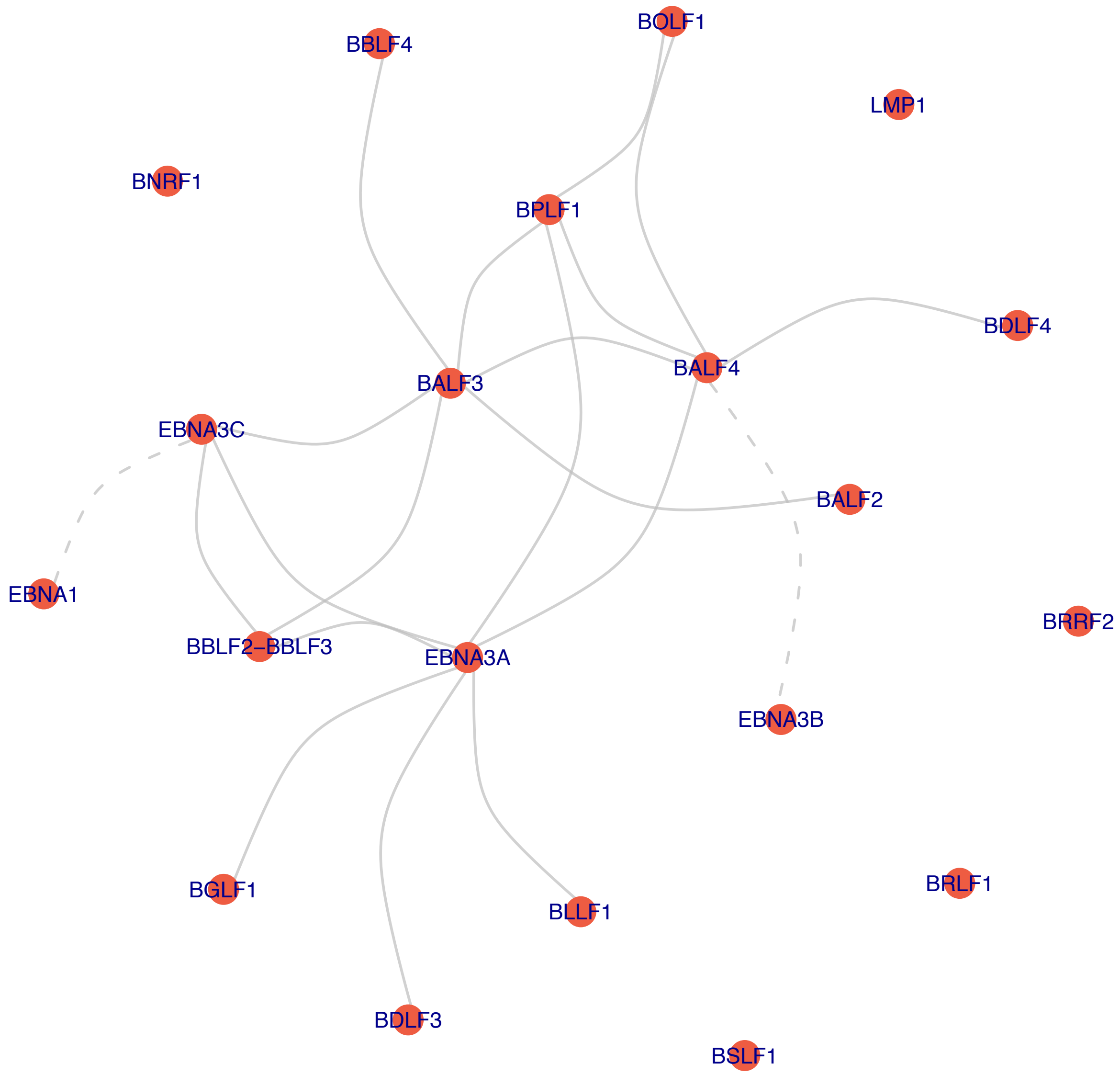

Suppl. Figure 9: Protein-protein interactions only between top 25 Eigenvector centrality tanked nodes of the gene network. Single nodes do not interact with any of the top 25 genes, but do have interactions with other EBV proteins. Interaction data is from Calderwood et al., 2007 and Fossum et al., 2009 based on Yeast two hybrid screenings. Low confidence interactions (Calderwood et al. 2007) are shown as dashed lines.

Supplementary methods

Selection of sequences for data set

The alignment published in Correia et al. 2018 (minus type 2 sequences and identical sequences) was extended with a selection of 10 sequences published in Chen et al. 2018 and Hui et al. 2018.
In these two publications, in total 206 novel genomes were published from healthy volunteers and NPC patients from Hong Kong, EBVaGC from Southern China, as well as one cell line from Korea. We based the selection on a NJ tree (K80 distance) of these novel Asian genomes and selected either representatives of bigger, similar clades as well as individual sequences with long branches, in order to capture those that add diversity to the data set (suppl. fig. 10).

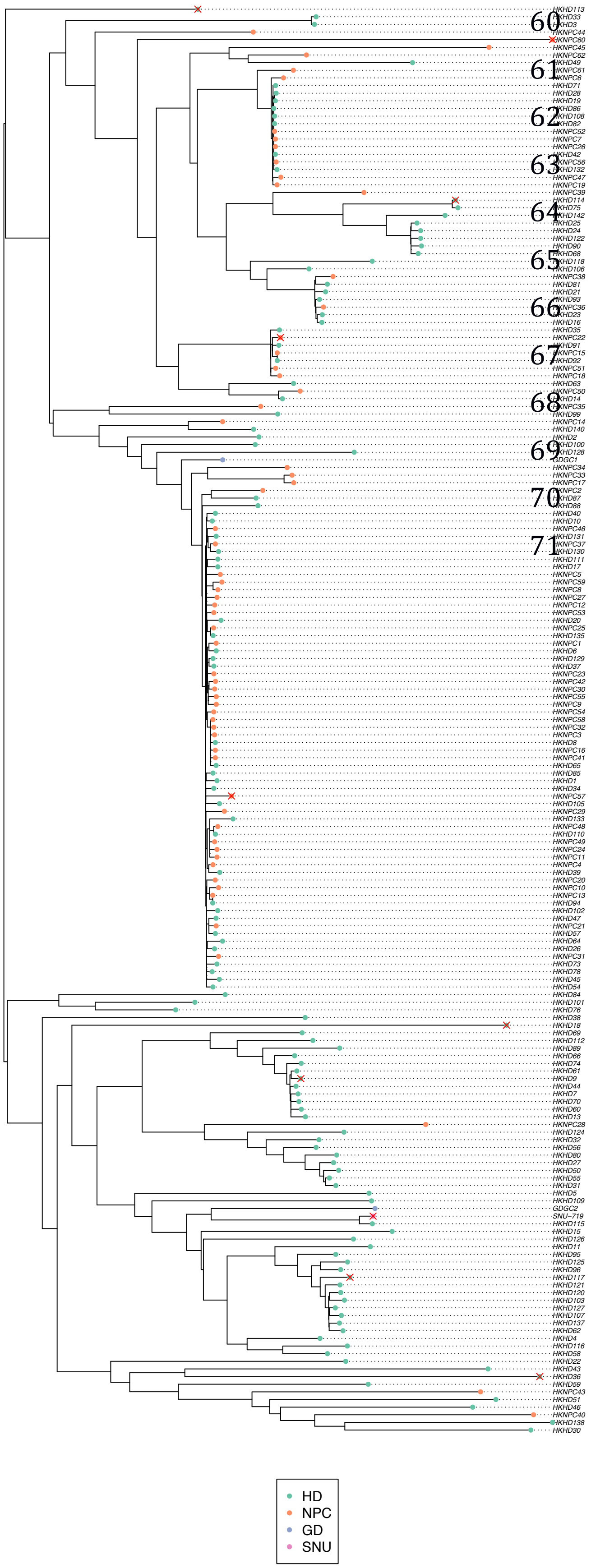

Suppl. Figure 10: NJ tree of sequences from Chen et al. 2018 and Hui et al. 2018. The sequences marked with a red X were selected to extend the data set from Correia et al. 2018.

HD: Healthy from Hong Kong, NPC: NPC patient from Hong Kong, GD: GC from Guandong, China, SNU: Korean GC cell line.
